# Balancing omega-6 and omega-3 fatty acids in ready-to-use therapeutic foods (RUTF)

**DOI:** 10.1186/s12916-015-0352-1

**Published:** 2015-05-15

**Authors:** J Thomas Brenna, Peter Akomo, Paluku Bahwere, James A Berkley, Philip C Calder, Kelsey D Jones, Lei Liu, Mark Manary, Indi Trehan, André Briend

**Affiliations:** Division of Nutritional Sciences, Savage Hall, Cornell University, Ithaca, NY 14850 USA; Valid Nutrition, Cuibín Farm, Derry Duff, Bantry Co., Cork, Republic of Ireland; Valid International, 35 Leopold Street, Oxford, OX4 1TW UK; KEMRI-Wellcome Trust Research Programme, Kilifi, 230-80108 Kenya; Nuffield Department of Clinical Medicine, Centre for Tropical Medicine & Global Health, University of Oxford, Old Road Campus, Roosevelt Drive, Oxford, OX3 7FZ UK; Faculty of Medicine, University of Southampton, Institute of Developmental Sciences Building (MP887), Southampton General Hospital, Tremona Road, Southampton, SO16 6YD UK; Centre for Global Health Research and Section of Paediatrics, Imperial College, Norfolk Place, London, W2 1PG, UK; Department of Pediatrics, Washington University School of Medicine, 660 S. Euclid, Campus Box 8116, St. Louis, MO USA; University of Malawi College of Medicine, P/Bag 360 Chichiri, Blantyre, 3 Malawi; Department for International Health, University of Tampere School of Medicine, FIN-33014 Tampere, Finland; Department of Nutrition, Exercise and Sports, Faculty of Science, University of Copenhagen, Rolighedsvej 30, DK-1958 Frederiksberg, Denmark

**Keywords:** Docosahexaenoic acid supplementation, High oleic peanuts, Omega-3 fatty acids, Ready-to-use therapeutic foods, Severe acute malnutrition

## Abstract

Ready-to-use therapeutic foods (RUTFs) are a key component of a life-saving treatment for young children who present with uncomplicated severe acute malnutrition in resource limited settings. Increasing recognition of the role of balanced dietary omega-6 and omega-3 polyunsaturated fatty acids (PUFA) in neurocognitive and immune development led two independent groups to evaluate RUTFs. Jones et al. (BMC Med 13:93, 2015), in a study in *BMC Medicine*, and Hsieh et al. (J Pediatr Gastroenterol Nutr 2015), in a study in the *Journal of Pediatric Gastroenterology and Nutrition*, reformulated RUTFs with altered PUFA content and looked at the effects on circulating omega-3 docosahexaenoic acid (DHA) status as a measure of overall omega-3 status. Supplemental oral administration of omega-3 DHA or reduction of RUTF omega-6 linoleic acid using high oleic peanuts improved DHA status, whereas increasing omega-3 alpha-linolenic acid in RUTF did not. The results of these two small studies are consistent with well-established effects in animal studies and highlight the need for basic and operational research to improve fat composition in support of omega-3-specific development in young children as RUTF use expands.

Please see related article: http://www.biomedcentral.com/1741-7015/13/93

## Background

Ready-to-use therapeutic foods (RUTFs) form the basis of the nutritional management of uncomplicated severe acute malnutrition (SAM), administered to millions of children worldwide every year [1]. RUTFs are intended as the sole food for several weeks during the rapid catch-up growth phase of treatment. Therefore, their nutritional composition must be complete and appropriate to support all aspects of growth and development.

The conventional recipe for RUTFs leads to a high energy density food made with a peanut base with added powdered milk, sugar, and fat, with 45% to 60% of the energy derived from fat. Commodity peanuts and predominant vegetable oils from which RUTFs are commonly made contain a high omega-6 linoleic acid (LA) content relative to essential fatty acid requirements and negligible omega-3 alpha-linolenic acid (ALA) as sources of omega-6 and omega-3 fatty acids, respectively. LA and ALA are the dominant forms of the two polyunsaturated fatty acid (PUFA) families acquired from plant foods, particularly vegetable oils. Their primary function is to serve as substrates for endogenous metabolism, which converts them to long chain PUFAs (LC-PUFA). Best known among these are omega-6 arachidonic acid (AA) and omega-3 eicosapentaenoic acid (EPA) and docosahexaenoic acid (DHA). Omega-6 LA and AA are seldom, if ever, limiting in the diet of otherwise well-nourished free living humans, while EPA and particularly DHA levels are known to be limiting from human studies that show DHA supplements improve status and function. Neural tissue membranes are particularly rich in DHA, accumulating perinatally, and both EPA and DHA have roles in immune function and modulation of inflammation. They can be consumed through foods of marine origin (e.g., fish, shellfish), but these are often expensive and/or prone to rapid spoilage, a property incompatible with the RUTF requirement of a long shelf-life under ambient environmental conditions.

Scores of studies show that developing animals, deprived of omega-3 fatty acids using peanut and similar omega-3 fatty acid-deficient oils during development, grow normally but have functional deficits. These include poor maze navigation performance, aggression, poor impulse control, and poor balance, to name a few, as well as a myriad of biochemical deficits [2]. This is due, in part, to replacement of the major structural fatty acid in the brain, omega-3 DHA, by an abnormal amount of the analogous omega-6 fatty acid docosapentaenoic acid, leading to neurocognitive deficits [2]. RUTFs do have marginal amounts of omega-3 ALA delivered by including an oil such as soybean or rapeseed oil with small amounts of ALA; normally, such oils contain more omega-6 LA and thus result in an RUTF which is out of balance with respect to the child’s sole nutritional source of the two essential fatty acid families. Beyond this, effects of the tissue omega-6-omega-3 balance on inflammation and blood clotting are well recognized, and recent work has implicated them in pain sensitivity, which likely has repercussions in psychological well-being [3,4].

While RUTFs are recognized as the major contributor to children’s recovery from SAM, increasing recognition of support of normal development has led to more careful consideration of oil composition.

## Balancing polyunsaturated fatty acids

Ample clinical evidence from well-nourished infants in developed countries is available to recommend an optional, adequate intake level of omega-3 DHA in infant artificial formulas to support development of neural tissue [5], confirming the idea that a properly functioning brain cannot be built without a dietary supply of omega-3 fatty acids and balanced omega-6 fatty acids, especially LA [6]. Unlike other omega-3 LC-PUFAs, circulating DHA levels in adults are unresponsive to supplementation with any precursor, including ALA, although some response has been observed in young infants [7].

Two recently completed clinical studies were first attempts to address the balance of omega-6 and omega-3 fatty acids in RUTFs, with primary endpoints being circulating LC-PUFA status. In a study in *BMC Medicine*, Jones et al. [8] increased omega-3 fatty acids against a background of constant omega-6 LA in two different ways. A test RUTF with 4.7-fold more omega-3 ALA, the DHA precursor from flaxseed oil (F-RUTF, Table 1), was provided to one experimental group; a second group received that test RUTF, along with EPA-DHA-containing fish oil (FFO-RUTF) from capsules [8]. Circulating DHA successfully increased with fish oil supplementation, as expected from many trials with preformed DHA.

**Table 1:** **Comparison of the plasma phospholipid fatty acid changes for treatments that exclusively increase ALA (Jones et al.** [8]**) vs. those that decrease LA and increase ALA (Hsieh et al.** [9]**)**

		**Intake (% wt)**		**Plasma phospholipid DHA (% wt)**			
**Jones et al.** [8]**, BMC Medicine 2015**							
**n = 20 per group**		LA	ALA	Basal	28 d	Diff (%)	
Control	S-RUTF	14.9	1.3	2.51	2.23	−11%	n.s.
	F-RUTF	14.4	6.2	2.63	2.08	−21%	n.s.
**Hsieh et al.** [9]**, JPGN 2015**							
**Control, n = 38; HO-RUTF, n = 43**							
Control	C-RUTF	21.3	0.4	3.24	2.43	−25%	*P* = 0.04
	HO-RUTF	13.1	13.1	2.84	2.96	4%	n.s.

Jones et al. [8]: From entry DHA drops non-significantly from baseline with a 4.7-fold increase in ALA from flax with no change in LA for both standard (S) and flax oil supplemented (F) RUTF. Hsieh et al. [9]: DHA drops dramatically in control (C); the drop in LA in high oleic (HO) prevents the decrease. Units are percent by weight of fatty acids.

In another study, Hsieh et al. [9] reduced omega-6 LA and increased omega-3 ALA, facilitated in part using high oleic peanuts to yield 13% of the total fatty acids from each of LA and ALA (HO-RUTF), with a similar total PUFA content to the control (C-RUTF) [9]. The two studies had a different ‘standard’ RUTF used as control, with a higher LA and a lower ALA content in the study from Hsieh et al. [9] compared to the study from Jones et al. [8]. The experimental RUTFs in both studies had similar LA contents (13.1% vs. 14.4%) but differed in the ALA content (13.1% vs. 6.2%; Table 1).

Both studies reported plasma phospholipid DHA, a form that is receptor-transported into the brain, at 28 days of treatment. The Jones et al. [8] study showed that both control and F-RUTF decreased DHA status, by −11% and −21%, respectively, although these differences from baseline were not significant. Consistent with this observation, Hsieh et al. [9], using a larger sample size, showed a significant decrease in their control group (−25% in DHA with C-RUTF). This decrease was avoided in their experimental group (HO-RUTF, +4% increase, not significant), indicating that the form of DHA transported most efficiently to the brain remained stable through the initial recovery period.

## Interpretation in the context of LC-PUFA nutrition

The results of the two studies are consistent with the hypothesis that standard RUTF results in a decline in DHA status. The two experimental groups were very similar in their LA content and differed only in ALA content. However, the difference in ALA between the two experimental diets (13.1% vs. 6.23%) is unlikely to explain the results as human and animal studies show that no amount of any omega-3 precursor – ALA, stearidonic acid, EPA, or omega-3 docosapentaenoic acid – improves DHA status [7]. Differences in other nutrients may play a role, including mineral status, which influences function of the iron-containing desaturases required for endogenous synthesis of DHA [10,11].

Reduction of omega-6 LA intake, as in the experimental group in the Hsieh et al. [9] study, has been observed to increase DHA status in at least three human studies [12] as expected from decades of animal studies. The intake range for effects is not well established in humans, especially malnourished children, and it is likely to differ based on age and physiological state, among other factors. Importantly, the amount of omega-6 LA required to prevent frank deficiency symptoms in otherwise well-nourished infants is less than 1% of energy but with seed oils it is often more than 10-fold this amount.

Both studies raise and attempt to address the serious issue of omega-3 adequacy in RUTF for severely malnourished children. Both studies demonstrated the safety and acceptability of the experimental RUTFs. Neither study was designed to identify a formulation producing optimal DHA status or measured neurodevelopment. It has long been known that omega-6 grows brawn, while omega-3 grows brains [6]. Although neither study was powered to detect effects on recovery from SAM, there is every reason to believe that oil formulations altering the relative proportions of the major fatty acids LA, ALA, and oleic, among others, will support energy needs.

## Conclusions

These studies both point to the vital need for trials of RUTFs with balanced PUFA content in multiple locations using a harmonized methodology, assessing linear growth, neurodevelopment, and infectious disease episode endpoints. LA reduction well below 13% can be achieved with high oleic, low LA peanuts. Novel sources of preformed DHA as supplements should also be considered, but if included directly in RUTF are likely to increase cost substantially and/or reduce shelf-life; the study by Jones et al. [8] highlighted a potential issue with the shelf-life for RUTFs with an elevated ALA content, a concern with any strategy that raises PUFA levels. In contrast, high oleic, low LA oils were developed to be more stable than their conventional higher PUFA content counterparts.

Until such studies are available, the need for further improvements should not distract from the fact that RUTFs are currently a life-saving intervention despite concerns over the decline in DHA status. Expanded coverage and improved delivery of therapeutic feeding services is a vital need.

## Abbreviations

AA: Arachidonic acid
ALA: Alpha-linolenic acid
DHA: Docosahexaenoic acid
EPA: Eicosapentaenoic acid
LA: Linoleic acid
LC-PUFA: Long chain PUFAs
PUFA: Polyunsaturated fatty acid
RUTFs: Ready-to-use therapeutic foods
SAM: Severe acute malnutrition
